# Aggregation of Thermoresponsive Polymethacrylates in a Dulbecco’s Modified Eagle Medium and Its Salts

**DOI:** 10.3390/polym15173587

**Published:** 2023-08-29

**Authors:** Łukasz Otulakowski, Barbara Trzebicka

**Affiliations:** Centre of Polymer and Carbon Materials, Polish Academy of Sciences, M. Curie-Skłodowskiej 34, 41-819 Zabrze, Poland

**Keywords:** thermoresponsive polymers, PNIPAM, poly(oligo ethylene glycol methacrylate), DMEM, salts, amino acids, aggregation

## Abstract

The thermal behavior and aggregation process of the poly(N-isopropyl acrylamide), poly[oligo(ethylene glycol) methyl ether methacrylate], and poly[(2-hydroxyethyl methacrylate)-co-oligo(ethylene glycol) methyl ether methacrylate] thermoresponsive polymers were studied in a commonly used Dulbecco’s Modified Eagle Medium (DMEM) cell culture medium and solutions of its individual components in the same concentration as found in DMEM. All studied copolymers exhibited an unexpected transmittance profile in the DMEM. During heating above the cloud point temperature (T_CP_), the polymers additionally aggregated, which led to the formation of their precipitates. The behavior of the polymers was further studied to evaluate how individual salts affected the transition temperature, size (D_h_), and stability of the polymer particles. Organic additives, such as amino acids and glucose, had a significantly lesser impact on the thermoresponsive aggregation of the polymers than inorganic ones. Changes to the T_CP_ were small and the formation of precipitates was not observed. The presence of small amounts of amino acids caused a decrease in the polymer aggregate sizes. Obtained results are of utmost importance in thermoresponsive drug nanocarrier studies.

## 1. Introduction

Thermoresponsive polymers undergo reversible soluble-insoluble phase transitions during heating. Polymers exhibiting a lower critical solution temperature (LCST) in dilute aqueous solutions above a certain temperature called the cloud point temperature (T_CP_) form well-defined spherical particles (mesoglobules) [1,2,3]. The size of mesoglobules depends on a number of factors, such as the polymer molar mass [4], solution concentration [5], heating protocol [6], and the presence of additives, i.e., salts [7] and surfactants [8,9,10]. Among the polymers that undergo a thermal transition during heating, the most extensively studied are the homo- and copolymers of *N*-isopropylacrylamide (NIPAM) [11,12,13,14], oxazolines [15,16,17,18], and oligo(ethylene glycol)methacrylates (OEGMA) [19,20,21,22].

The high potential of thermoresponsive polymers as drug carriers derives from the ability to control their thermal aggregation and the size of the particles formed [23,24]. An important aspect in the preparation of nanocarriers from these polymers is their aggregation and easy stabilization of the obtained particles [25,26,27,28]. In this regard, understanding the behavior of thermoresponsive polymers in solutions containing components present in the biological medium is essential for the possibility of their use for medical purposes.

Studies of the influence of additives on macromolecules in solution were first conducted in 1888 by Hofmeister [29]. All salts were divided into one of the two groups, kosmotropic and chaotropic salts, which produce “salting in” and “salting out” effects, respectively. The presence of salts changes the hydration sphere that surrounds the substance molecules dissolved in a solution and, as a result, changes their solubility. Traditionally, the relative effects of anions present in solution on the physical behavior of solutes have been ranked according to the Hofmeister series: CO_3_^2−^  >  SO_4_^2−^  >  S_2_O_3_^2−^  >  HPO_4_^2−^  >  H_2_PO_4_^−^  >  F^−^  > Cl^− ^ >  Br^−^ > NO_3_^−^  > I^−^  > ClO_4_^−^ > SCN^−^. Numerous reports in the literature have indicated that the presence of salts in the solution of a thermoresponsive polymer may alter the transition temperature at which phase separation occurs [30,31,32]. The effect is dependent on the affinity of the salt with water and the polymer molecules. Salts in a water solution can influence the hydrogen bonding network between water molecules and polymer chains, which affects their stability in the solution. It has been shown that chaotropic salts would allow water molecules to favorably interact with the polymers and stabilize the intramolecular interactions within these macromolecules [33]. This effect known as “salting in” leads to an increase in the T_CP_. On the other hand, kosmotropic anions may lead to a “salting out” effect, and their presence disturbs the hydration sphere of the polymer chain, thereby decreasing the polymer solubility. Kosmotropic salts contribute to the stability of water–water interactions. Both “salting in” and “salting out” effects are enhanced by an increase in salt concentrations [34].

The influence of the salt addition on shifting the T_CP_ has previously been studied for poly(N-isopropylacrylamide) (PNIPAM) [35], poly(2-ethyl-2-oxazoline) [36], poly(2-n-propyl-2-oxazoline) [26], poly(2-isopropyl-2-oxazoline) [36], poly(oligo(ethylene glycol) methyl ether methacrylate [31], and others [37]. The aggregates of thermoresponsive polymers formed in the presence of salts during heating above the T_CP_ have only rarely been reported [38]. Although changes to the T_CP_ of thermoresponsive polymers in salt solutions have been investigated [7,30,35,36], there have been no studies on the effect of salts on the aggregation process and particle size and their formation. Studies of thermoresponsive polymers in salt solutions have frequently emphasized salts that are mostly irrelevant in biological systems [31,39].

Selianitis et al. [40] reported on the behavior of amphiphilic hyperbranched poly[oligo(ethylene glycol) methyl methacrylate)-co-poly(2-(diisopropylamino)ethyl methacrylate] in response to temperature and pH in different solutions. The authors observed the formation of more well-defined nanoparticles with narrower size distributions in phosphate-buffered saline (PBS) than in their dispersions in media with a pH of 7. The presence of salt in PBS solutions, together with the increase in temperature, appears to affect the structure of the aggregates in a different way than solutions with a similar pH that contain smaller amounts of salt.

The influence of added substances has been studied more extensively for the commercially available Pluronic^®^ and Tetronic^®^ ethylene oxide-propylene oxide block copolymers. It has been revealed that due to their amphiphilic structure, these copolymers formed micelles in solution but also underwent subsequent thermal aggregation above the critical temperature [41]. Patidar et al. [42] studied the influence of several substances, including salts, amino acids, urea, and sugars on the T_CP_ of Pluronic^®^ and Tetronic^®^. The dependencies observed for the salt solutions were in agreement with those proposed in the Hofmeister series. In all studied cases, the presence of amino acids led to a decrease in the T_CP_. It was suggested that more than one effect played a role in the interactions of additives with polymers, i.e., acidic/alkaline interactions or their aqueous solubility, the solution pH, and the hydrophobic/hydrophilic balance. Extensive studies of amphiphilic poly(ethylene oxide)-poly(propylene oxide)-poly(ethylene oxide) (PEO–PPO–PEO) copolymers have been conducted by Nandni et al. [43]. The authors studied the addition of different biomolecules, including amino acids, dipeptides, and amino alcohols, to the solutions of PEO–PPO–PEO copolymers and determined the influence of additives on the measured cloud point. Amino acids were found to promote the dissolution of polymer micelles and cause a reduction in the T_CP_.

The aforementioned studies focused on cloud points but did not consider the changes to aggregate sizes under the influence of small substances with temperature changes. The stability of dispersions with additives was not revealed.

Relatively few studies have focused on studying thermoresponsive polymers in media of physiological importance or the presence of biomolecules. Indeed, research has commonly been performed on solutions containing only one additive at a time. In most cases, although the authors used in their studies both thermoresponsive polymers and the DMEM [44,45], they did not focus on the problem of polymer behavior in the DMEM. The only exception we found was the work of Takezawa et al. [46] in which the authors described a drop in the T_CP_ for PNIPAM from around 32 °C in water to 28–29 °C in the DMEM.

The DMEM used in in vitro studies of drugs and nanocarriers is a complicated mixture of salts and amino acids of biological importance supporting the growth of many different mammalian cells. Cells that can be successfully cultured in the DMEM include primary fibroblasts, neurons, glial cells, human umbilical vein endothelial cells (HUVECs), and smooth muscle cells, as well as cell lines such as HeLa, 293, Cos-7, and PC-12. Therefore, the problem of the behavior of thermoresponsive polymers in this medium is becoming increasingly important.

Aggregation and its reversibility are fundamental factors for the possible biomedical application of thermoresponsive polymers [47,48]. In this work, we report on the behavior of common thermoresponsive polymers, such as PNIPAM, a copolymer of poly[(2-hydroxyethyl methacrylate)-*co*-oligo(ethylene glycol) methyl ether methacrylate] (P(HEMA_10_-*co*-OEGMA_90_), and poly[oligo(ethylene glycol) methyl ether methacrylate] (POEGMA) in the DMEM and solutions containing its most important components. Aqueous solutions of the copolymers containing one of the DMEM components in the same concentration as in the DMEM were also measured. The additives, six substances of inorganic origin and six of organic origin, were selected, with concentrations exceeding 0.1 g/L in the DMEM. Transmittance and light scattering studies of the solutions of thermoresponsive polymers heated and incubated above the phase transition temperature showed the influence of additives on the T_CP_, the sizes of aggregates, and their stability. Our results should help to draw attention to the unexpected behavior of thermoresponsive polymers and reveal the need for research in physiological environments, especially on the use of these polymers for medical purposes.

## 2. Materials and Methods

### 2.1. Materials

Dulbecco’s Modified Eagle Medium (DMEM) (D5030), sodium chloride (NaCl), sodium bicarbonate (NaHCO_3_), potassium chloride (KCl), calcium chloride (CaCl_2_), monosodium phosphate (NaH_2_PO_4_), copper(II) sulfate (CuSO_4_), glucose, L-glutamine, L-lysine × HCl, L-isoleucine, L-leucine, L-tyrosine × 2Na, 2-hydroxyethyl methacrylate (HEMA, ≥99%), oligo(ethylene glycol) methyl ether methacrylate (OEGMA_300_, M_n_ = 300 g/mol), and PNIPAM were purchased from Sigma-Aldrich (Germany) and used as received. The P(HEMA-*co*-OEGMA) with 90% mol of HEMA as well as homopolymers of OEGMA were synthesized at the Centre of Polymer and Carbon Materials of the Polish Academy of Sciences via atom transfer radical polymerization (ATRP) as described in [49]. The polymer characterization is summarized in Table 1.

The water used to form the solutions was purified using a commercial ion exchange system (Hydrolab, Poland) and filtered twice through a polytetrafluoroethylene (PTFE) filter (0.2 μm) (Merc Millipore, Burlington, MA, USA).

### 2.2. Methods

#### 2.2.1. Dulbecco’s Modified Eagle Medium and Salt Solution Preparation

The DMEM was prepared through the direct dissolution of powder provided by the manufacturer (portion for 1 L of solution) in 0.5 L of water. The salts studied in the individual solutions were chosen based on their concentration in the DMEM (Table 2). A full list of DMEM compositions is presented in the Supplementary Information (SI; Table S1). Individual solutions were prepared by dissolving an appropriate amount of low molar mass components in water according to their concentration in the DMEM. The solutions were placed on a mechanical shaker overnight to ensure the complete dissolution of the salt.

#### 2.2.2. Polymer Solution Preparation

Each studied polymer was dissolved directly in water at a concentration of 1 g/L. To ensure the complete dissolution of the polymers, the solutions were placed in a refrigerator at 4 °C overnight. Following the dissolution, 2.5 mL of polymer solutions were diluted with 2.5 mL of DMEM and single salt solutions (concentration double that of the concentration required). The polymer concentration in all the solutions was 0.5 g/L and the concentrations of salt were as specified in the DMEM.

#### 2.2.3. Turbidity Measurements

The T_CP_ values in the prepared solutions were determined using a SPECORD 200 PLUS UV-Vis spectrophotometer (Analytik, Jena, Germany) with a Peltier temperature-controlled cell holder. The samples were analyzed at a constant transmittance wavelength (λ = 550 nm). The polymer solutions were heated from 15 °C to a temperature around 25 °C greater than the T_CP_ at a heating rate of 1 °C/min. Then, the samples were cooled down to 15 °C at a cooling rate of 1 °C/min. The T_CP_ value was determined by the temperature at which the second derivative of the transmittance versus temperature plot crossed 0.

#### 2.2.4. Characterization by Dynamic Light Scattering

Dynamic light scattering (DLS) measurements were obtained by abruptly heating 1 mL of the samples to temperatures above the transition temperature and then stabilized for 15 min and measured every 15 min for 5 h. A Zetasizer ZS90 (Malvern Instruments, Malvern, Worcestershire, UK) analyzer equipped with a helium-neon laser with a wavelength of λ = 632.8 nm and a power of 4 mW was used. The correlation curve was developed using the CONTIN algorithm. The dispersion index (PDI) was obtained according to the equation $\mu_{2}/{\bar{\Gamma }}^{2}$, where $\bar{\Gamma }$ is the average relaxation rate and *µ*_2_ is its second moment. The measurement was repeated three times for each sample. The acquired data were processed with the Zetasizer software version 6.32 (Malvern Instruments, Malvern, Worcestershire, UK).

## 3. Results

The PNIPAM, OEGMA, and HEMA copolymers are being considered as possible materials for drug delivery due to their ability to be customized, their responsiveness to thermal stimuli within the range of a human body’s temperature, and their biocompatibility.

Poly(*N*-isopropylacrylamide), whose properties have previously been studied and described in detail [13,50,51], is frequently treated as a model thermoresponsive polymer (T_CP_ = 32 °C for c = 1 g/L). *N*-isopropylacrylamide is also the foundation for many thermoresponsive copolymers. Some of these copolymers have been conjugated with bioactive species, such as peptides, proteins, and drugs [52,53,54,55,56]. The use of ethylene glycol methacrylates in copolymerization with other monomers allows for the delivery of macromolecules with a wide range of transition temperatures [22,57]. The T_CP_ of OEGMA_300_ homopolymers in pure water is around 66.5 °C at a concentration of 2 g/L [58]. The biocompatibility of the oligo(ethylene glycol) pendant groups allows POEGMA-based materials to be used in biomedical fields [59]. In turn, P(HEMA_90_-OEGMA_10_) copolymers are thermoresponsive with a T_CP_ in water of around 23 °C for concentrations of 0.5 g/L [49]. PHEMA homopolymers are insoluble in water and form a gel at degrees of polymerization exceeding 45 because of strong interchain hydrogen bonding [60]. Copolymers based on HEMA have good hemo- and biocompatibility. Moreover, they are nontoxic, which is desirable for applications in medicine [61]. The polymers selected for this study had 90 mol% of HEMA and 10 mol% of OEGMA in their chains. This copolymer composition allowed for their solubility in cold water.

As discussed in the Introduction, the properties of thermoresponsive polymers in aqueous solutions were the subject of many previous studies. However, their behavior in an environment similar to that found in the human body or cell culture media has only been briefly described, and the details of their aggregation processes and aggregate formation have not been considered.

The thermal behavior of thermoresponsive PNIPAM, P(HEMA_90_-*co*-OEGMA_10_), and POEGMA in the DMEM and solutions containing individual DMEM components was investigated using ultraviolet-visible (UV-vis) spectroscopy measurements during heating and cooling over temperature ranges well below and well above their T_CP_. The transmittance changes were measured for solutions with a total polymer concentration of 0.5 g/L.

The DLS studies were conducted for the samples abruptly heated to temperatures exceeding the T_CP_. The copolymer dispersions were incubated at elevated temperatures for 5 h. During this time, the sizes of the aggregates and the solution scattering intensity were monitored.

### 3.1. Thermal Behavior of (Co)Polymers in Water

**Figure 1:**
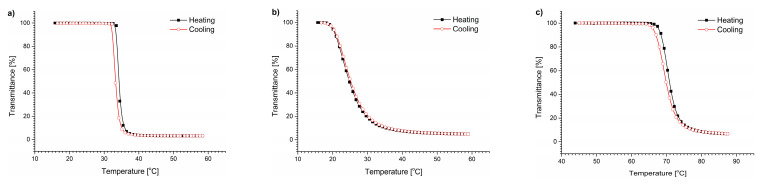
Transmittance vs. temperature changes during heating and cooling of (**a**) PNIPAM, (**b**) P(HEMA_90_-OEGMA_10_), and (**c**) POEGMA solutions in water, c = 0.5 g/L.

In the first step, the polymers were tested in pure water. The transmittance vs. temperature curves for the polymers in water are sigmoidal (Figure 1). The cooling curves indicate a full reversibility of the thermal aggregation. The T_CP_ values determined for the polymers in water are presented in Table 3. As expected, the obtained T_CP_ values are in good agreement with those reported in the literature [49,62,63].

The next step involved studying the stability of the polymer aggregates through light scattering measurements. The solutions of copolymers were abruptly heated to 20–25 °C above their T_CP_. As reported in the literature, a sharp increase in the polymer solution temperature led to the formation of particles smaller than those formed during slow heating [6,22,64]. Following the abrupt heating, the suspensions were incubated for 5 h. The diameters of the mesoglobules are listed in Table 3 (the sizes provided are the highest values detected in every sample). In all cases, the sizes and light scattering intensity of the particles formed in water were stable over the 5 h of incubation (Figure S1) without traces of secondary aggregation or precipitation of particles. The particle size distributions are presented in Figure S2a–c.

### 3.2. Thermal Behavior of (Co)Polymers in the DMEM

**Figure 2:**
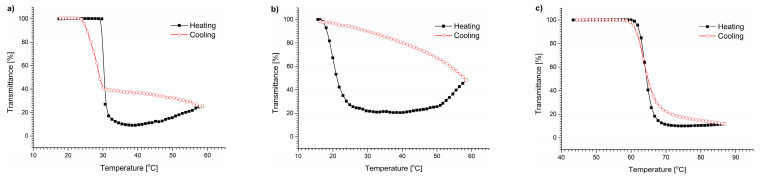
Transmittance vs. temperature changes during heating and cooling of (**a**) PNIPAM, (**b**) P(HEMA90-OEGMA10), and (**c**) POEGMA solutions in DMEM, c = 0.5 g/L.

The transmittance changes of the polymer/DMEM solutions during heating and cooling are shown in Figure 2.

The dispersions in the UV-Vis experiments were not allowed to anneal at high temperatures for a long time, and instead, they were cooled immediately after reaching the maximal temperature of the measurement. The transmittance curves of the polymers upon cooling at the same rate as heating (1 °C/min) did not follow the profile of the heating curves. For PNIPAM and P(HEMA_90_-OEGMA_10_), the transmittance curves were not sigmoidal. When the temperatures increased, the transmittance curves reached their minimum before starting to rise. The heating of the solutions to temperatures above the temperature of the minimum transmittance led to the subsequent polymer precipitation. The dispersion slowly became less opaque, indicating a decrease in the particle number in the sample volume that was “visible” to the UV beam. A similar behavior, i.e., the precipitation of P(HEMA-OEGMA), has also been observed in PBS [49,65]. The transmittance curve of the POEGMA hardly differed from the sigmoidal dependence typical for thermoresponsive polymers in water. Lowering the temperature to room temperature increased the transmittance to the initial value (100%).

We have evidenced that the cooling of the solutions immediately after passing through the T_CP_ resulted in a fully reversible transition. To induce additional aggregation, the dispersions must be heated to temperatures exceeding that of the minimal transmittance. These data indicate that polymers undergo additional aggregation during heating. The aggregation process that leads to the formation of precipitates is hardly reversible. The precipitates remain stable during cooling and do not dissolve. The dissolution of the precipitates requires their prolonged incubation at temperatures well below T_CP_ (+4 °C).

The T_CP_ of the copolymers in the DMEM presented in Table 3 was determined as the temperature at which the second derivative transmittance versus the temperature plot crossed zero during heating. The transition temperatures of the polymers in the DMEM were shifted toward lower temperatures compared with the solutions in pure water (Table 3). The highest shift of 6.1 °C was observed for POEGMA and the smallest (1 °C) for PNIPAM. The lowering of the T_CP_ indicates that the water–polymer interactions in the DMEM were replaced to some extent by salt interactions with water, causing a “salting out” effect.

**Figure 3:**
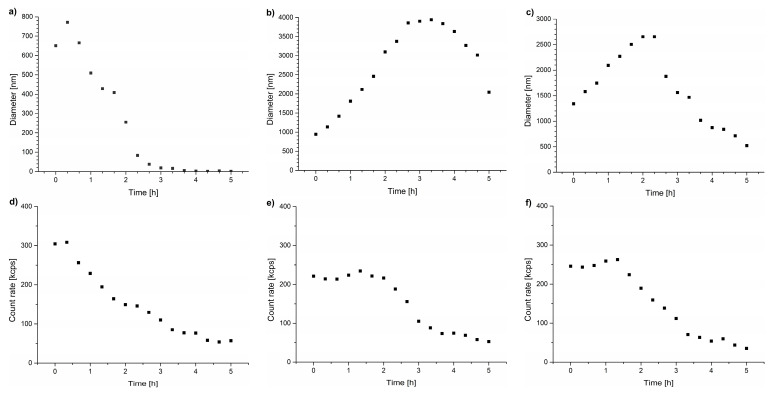
Hydrodynamic diameters of particles vs. time of incubation in DMEM for (**a**) PNIPAM, (**b**) P(HEMA_90_-OEGMA_10_), and (**c**) POEGMA, and scattered light intensity for (**d**) PNIPAM, (**e**) P(HEMA_90_-OEGMA_10_), and (**f**) POEGMA.

The light scattering intensities of the samples were measured for 5 h to follow the temporal changes of particle sizes during the annealing of the dispersions at temperatures above T_CP_ (Figure 3). The maximum particle diameters observed during the annealing process are presented in Table 3, and particle size distributions at this point are provided in Figure S2d–f. As can be seen, size distributions for particles formed during annealing in the DMEM are much broader than those in water and unsymmetrical with long tails toward higher sizes.

For all the studied copolymers, the particles formed in the DMEM were unstable, which was reflected by a decrease in their size and light scattering intensity caused by polymer aggregation over time and their precipitation from the solution (Figure 3). The DLS studies showed that even for the POEGMA polymers in the DMEM, where the transmittance curve was the most similar to that of water (Figure 2c), significant secondary aggregation occurred, leading to the formation of micrometer-sized particles that precipitated from the dispersions during the incubation. The observed curves confirm that the precipitation of polymeric particles occurred in all studied cases.

### 3.3. Influence of Individual DMEM Additives on the Thermal Behavior of (Co)Polymers

To determine which of the components was the most responsible for the precipitation of copolymers, all copolymers were studied in solutions containing only one component of the DMEM culture medium. Additives of the DMEM with concentrations exceeding 0.1 g/L were chosen. The transition temperature values and maximal sizes of the particles formed during incubation above the T_CP_ are given in Tables S2–S4.

#### 3.3.1. Influence of Individual DMEM Salts on the Thermal Behavior of (Co)Polymers 

Sodium chloride (NaCl) and sodium bicarbonate (NaHCO_3_) influenced the thermoresponsive behavior of the studied copolymers the most. These salts were found in the highest concentrations in the DMEM, i.e., 6.4 g/L and 3.7 g/L, respectively.

**Figure 4:**
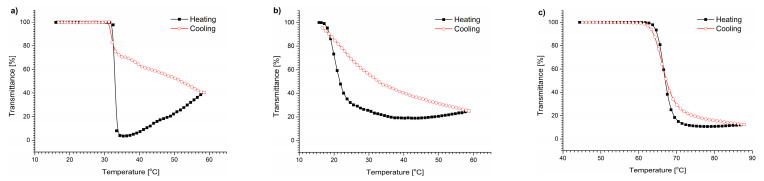
Transmittance vs. temperature changes during heating and cooling of (**a**) PNIPAM, (**b**) P(HEMA_90_-OEGMA_10_), and (**c**) POEGMA in solutions of NaCl.

The behavior of copolymers in the presence of NaCl (Figure 4) was similar to that in the DMEM itself (Figure 2). The aggregated polymer particles in the 6.4 g/L NaCl solution bound together forming bigger particles after passing the T_CP_, as was clearly visible in the UV-Vis traces of the PNIPAM and P(HEMA_90_-OEGMA_10_) dispersions.

The T_CP_ of the solutions was shifted toward temperatures lower than that of water in all cases. The shifts were only 1.2 °C for PNIPAM, 2.8 °C for P(HEMA_90_-OEGMA_10_), and 3.6 °C for POEGMA (Tables S2–S4). For the PNIPAM and P(HEMA_90_-OEGMA_10_) polymers, the shifts were similar to those observed in the DMEM. Only the T_CP_ of the POEGMA solution was shifted to temperatures higher than that of the DMEM (around 2.5 °C). As in the case of earlier studies in the DMEM, a slight hysteresis was present in the POEGMA curves obtained by UV-Vis measurements in this case, but the transmittance curve did not indicate precipitation. The transmittance curves of two other polymers in a NaCl solution indicated, as for the DMEM, the merging of structures formed above the T_CP_ and their precipitation from the solution, accompanied by an increase in the transmittance at higher temperatures.

**Figure 5:**
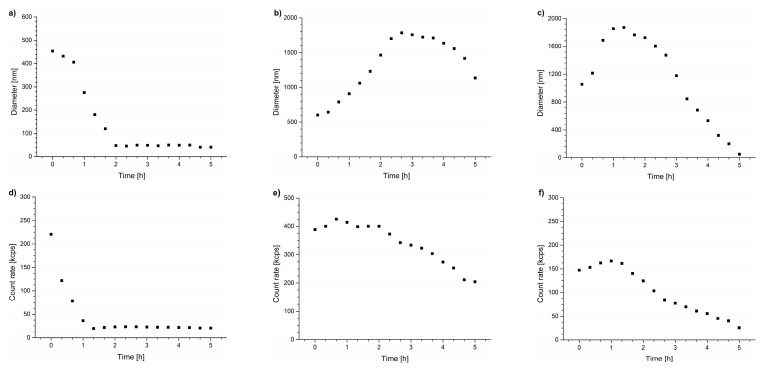
Hydrodynamic diameters of particles vs. time of incubation in a solution of NaCl for (**a**) PNIPAM, (**b**) P(HEMA_90_-OEGMA_10_), and (**c**) POEGMA, and scattered light intensity for (**d**) PNIPAM, (**e**) P(HEMA_90_-OEGMA_10_), and (**f**) POEGMA.

The precipitation phenomenon was evident from the DLS studies (Figure 5). Following the abrupt heating, all copolymers precipitated from the NaCl solution during incubation. The PNIPAM polymers underwent precipitation very rapidly; the diameters of the particles visible by light scattering decreased after only one hour of incubation (Figure 5a) and the scattered light intensity dropped to minuscule values, indicating a very small number of particles in the solution (Figure 5d). The sizes of the aggregates increased for other studied polymethacrylates during the first part of the incubation period (Figure 5b,c), while the scattering intensity curves did not change rapidly (Figure 5e,f). The POEGMA and P(HEMA_90_-OEGMA_10_) particles reached almost 2000 nm in size and, therefore, could not remain in colloidal suspension. Further incubation led to a decrease in aggregate sizes indicating the precipitation of the largest species, leading to a decrease in scattering intensity.

**Figure 6:**
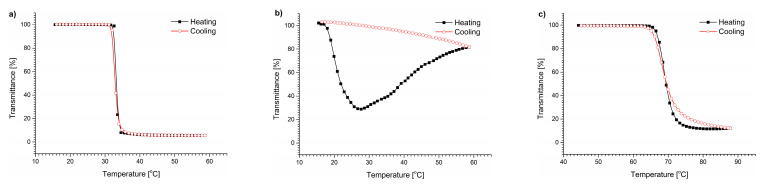
Transmittance vs. temperature changes during heating and cooling of (**a**) PNIPAM, (**b**) P(HEMA_90_-OEGMA_10_), and (**c**) POEGMA in solutions of NaHCO_3_.

The other studied salt that composed the DMEM was NaHCO_3_. The transmittance vs. temperature curves of the polymers in a 3.7 g/L NaHCO_3_ solution are presented in Figure 6.

The presence of NaHCO_3_ affected the PNIPAM polymers differently from what was observed for NaCl. The plot resembles the one obtained for PNIPAM polymer in water. The T_CP_ was shifted by only 1 °C to temperatures lower than those obtained for water but remained 4.6 °C higher than that of the DMEM (Table S2). The transmittance curves of the POEGMA are smooth and sigmoidal for both heating and cooling, and the UV-Vis traces nearly overlap. The T_CP_ was shifted by 1.4 °C to lower temperatures. The P(HEMA_90_-OEGMA_10_) transmittance curve is significantly different from that of water, and strongly resembles the one acquired from the DMEM (Figure 2b). The T_CP_ was 3 °C below that obtained for water (Table S3). Indeed, the heating and cooling traces do not overlap, and the cooling curve stays above the heating curve.

Dynamic light scattering studies provide further insights into polymer aggregation in NaHCO_3_ (Figure S3). The particles formed by PNIPAM in the solution of NaHCO_3_ (D_h_ = 336 nm) were around 120 nm larger than those formed in water (Table S2). Despite their larger size, the particles remained stable for the whole duration of the measurements and did not precipitate from the solution. The stability of the suspension was confirmed by its light scattering intensity, which also remained unchanged during the incubation process (Figure S3d).

The P(HEMA_90_-OEGMA_10_) polymers aggregated during incubation, forming particles that precipitated from the solution. The diameter of the P(HEMA_90_-OEGMA_10_) particles was around 500 nm at the beginning of the incubation period, but their merging led to 1900 nm sized particles which reluctantly precipitated from the solution causing a decrease in the scattered light intensity (Figure S3e). Despite the observed course of their UV-Vis curve, the POEGMA particles also underwent additional aggregation and precipitated from the dispersion during the incubation (Figure S3c). A steady and constant decrease in the scattered light intensity suggests that the largest species of aggregates were continuously precipitating from the solution. This strongly suggests that salt-mediated aggregation occurred, with salt participating in the formation of the aggregated structures.

**Figure 7:**
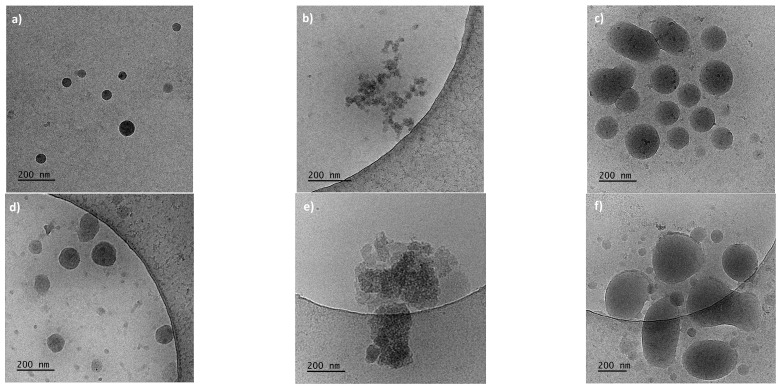
Cryo-TEM images of PNIPAM dispersions in (**a**) water, (**b**) NaCl, (**c**) NaHCO_3_, and P(HEMA_90_-OEGMA_10_) dispersions in (**d**) water, (**e**) NaCl, and (**f**) NaHCO_3_.

Cryo-TEM studies were conducted to gain further insight into the aggregation process in solutions containing additives. Microphotographs were taken of dispersions of PNIPAM and P(HEMA_90_-OEGMA_10_) in NaCl, NaHCO_3_, and in water (Figure 7). Samples were taken from above the precipitated part, so the biggest aggregates were not visible.

Cryo-TEM images revealed drastically different structures in studied dispersions depending on the additive. In water, well-defined dense spherical particles with sizes roughly corresponding to the DLS data were observed. In dispersions containing NaCl after incubation, no spherical particles but loose structures of indefinite shape were visible for both thermoresponsive polymers. In NaHCO_3_ dispersions, the morphology of aggregates in both cases is very similar. The aggregates with almost spherical shapes but with much more loose internal organization can be seen; almost see-through particles are clearly visible in Figure 7f.

#### 3.3.2. Influence of DMEM Amino Acids on the Thermal Behavior of (Co)Polymers 

The effect of amino acids hosted in the DMEM at concentrations greater than 0.1 g/L on the behavior of thermoresponsive polymer aggregation in their individual solutions was investigated. It should be emphasized that the concentration of amino acids in the DMEM was much lower than the concentrations of the salts in the abovementioned studies. The results of studies performed for amino acids are provided in Tables S2–S4. Three amino acids—L-lysine×HCl, L-tyrosine × 2Na, and L-glutamine—will be discussed below in more detail.

**Figure 8:**
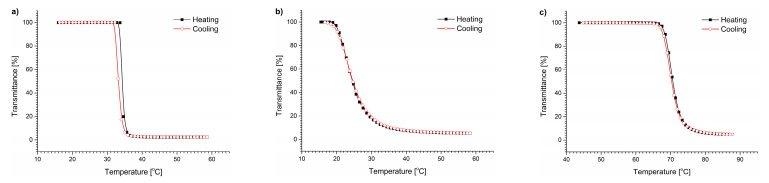
Transmittance vs. temperature plots for (**a**) PNIPAM, (**b**) P(HEMA_90_-OEGMA_10_), and (**c**) POEGMA in the solution of L-lysine×HCl.

As shown in Figure 8, all transmittance plots of the thermoresponsive polymers are smooth and sigmoidal in the presence of lysine hydrochloride at a concentration of 0.146 g/L. The T_CP_ of the copolymers did not change by more than 0.1 °C relative to pure water (Tables S2–S4).

**Figure 9:**
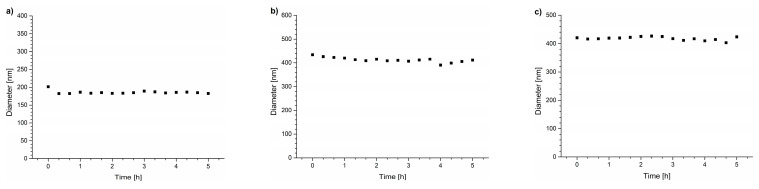

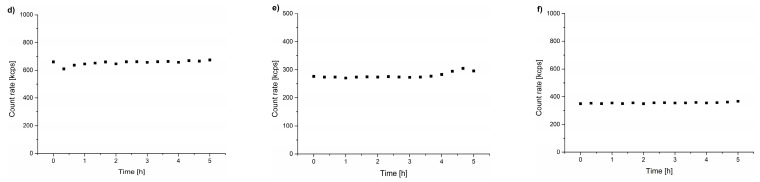
Hydrodynamic diameters of particles vs. time of incubation in a solution of L-lysine × HCl for (**a**) PNIPAM, (**b**) P(HEMA_90_-OEGMA_10_), and (**c**) POEGMA, and scattered light intensity for (**d**) PNIPAM, (**e**) P(HEMA_90_-OEGMA_10_), and (**f**) POEGMA.

Figure 9 shows the influence of lysine on the sizes and stability of particles formed by studied copolymers during the process of abrupt heating followed by incubation for 5 h. Although lysine hydrochloride did not change the T_CP_, it caused a decrease in the size of the mesoglobules formed by the polymer compared with those formed in water by 31, 41, and 52 nm for PNIPAM, P(HEMA_90_-OEGMA_10_), and POEGMA, respectively. The sizes remained unchanged during incubation. The light scattering intensity also remained constant (Figure 9d–f), indicating that the dispersion of particles was stable and no precipitation occurred.

Similar trends were observed for amino acids with the inorganic moiety L-tyrosine × 2Na (Figure S4). For solutions containing this additive, the T_CP_ increased slightly by 0.6, 0.9, and 0.5 °C for the PNIPAM, P(HEMA_90_-OEGMA_10_), and POEGMA polymers, respectively, relative to water (Tables S2–S4).

The presence of L-tyrosine × 2Na also led to a decrease in the size of the particles formed in the solution compared with water (Figure S5, Tables S2–S4). For PNIPAM and POEGMA, the particle diameters decreased by 55 and 45 nm, respectively. The most pronounced effect was visible for P(HEMA_90_-OEGMA_10_), where the diameter of aggregates decreased by over 120 nm. The scattering light intensity remained stable, indicating that no precipitation occurred during the incubation.

One noteworthy amino acid that occurred in the DMEM in its native form was L-glutamine. The transmittance plots of the polymer solution containing this amino acid are smooth and sigmoidal for all polymers (Figure S6). Its influence on the transition temperature was miniscule: +0.9 °C for PNIPAM, −0.7 °C for POEGMA, and almost no change for P(HEMA_90_-OEGMA_10_), relative to water (Tables S2–S4).

All polymers formed stable suspensions in the presence of L-glutamine; the sizes of the particles remained unchanged over time and they did not precipitate. In comparison with water, the diameter of the P(HEMA_90_-OEGMA_10_) particles increased by around 30 nm and that of the POEGMA particles by 100 nm. On the other hand, the size of the PNIPAM mesoglobules decreased by 44 nm (Figure S7).

The results obtained for the amino acids used as additives show the potential for their use in controlling the sizes of mesoglobules. Sodium dodecylsulfate (SDS) is currently one of the most commonly used substances serving to decrease the size of particles formed by thermoresponsive polymers. Studies in this field have been so far conducted for PNIPAM [9,10] and random copolyethers [66]. The disadvantage of this approach is the cytotoxicity of SDS. The replacement of SDS with substances of natural origin may be an important step in designing novel nanocarriers for biomedical usage.

#### 3.3.3. Influence of Glucose on the Thermal Behavior of Copolymers

One of the components of the DMEM culture medium that did not belong to the group of salts or amino acids was glucose. Glucose was an important component of the DMEM in which it occurred in high concentrations of 1 g/L. Our observations suggest that the influence of this sugar on the aggregation of the studied thermoresponsive polymers was not very significant. Indeed, the T_CP_ of the copolymers was slightly shifted positively for PNIPAM and P(HEMA_90_-OEGMA_10_) (+0.2 °C) and negatively for OEGMA (−0.5 °C) (Figure S8, Tables S2–S4). All transmittance curves are sigmoidal with no signs of hysteresis.

The particles formed in the glucose solution were stable during the whole duration of the measurements (Figure S9). The greatest influence on the sizes of the particles was observed for PNIPAM, with a particle size decrease of 30 nm in the glucose solution relative to water. The size of the aggregates for POEGMA in the glucose solution was slightly larger than in water (+10 nm). The diameters of the P(HEMA_90_-OEGMA_10_) particles remained practically unchanged. The scattering intensity also remained stable during the measurements.

## 4. Conclusions

Most scientific research in the literature has focused on the thermal behavior of thermoresponsive polymers in water. Studies of the aggregation of thermoresponsive polymers in solutions containing salts or solutions of physiological importance have mainly focused on the transition temperature. To fill these gaps, we investigated the temperature response of thermoresponsive polymethacrylates in the DMEM culture medium and solutions containing only one component of this medium. The transition temperatures in the DMEM decreased for all studied samples compared with these values in water. The heating of the solutions to temperatures exceeding that of the minimum transmittance led to subsequent polymer aggregation and precipitation. The precipitates remained stable during cooling and did not dissolve. The dissolution of the precipitates would require prolonged incubation in a fridge (+4 °C).

To determine which component was responsible for the polymer precipitation, studies were performed with individual DMEM ingredient solutions at concentrations corresponding to their concentrations in commercially available DMEM culture media.

Our study revealed that only some of the additives caused polymer precipitation and some (in studied concentrations) only caused an increase in the size of the aggregates. The presence of amino acids affected the size of the particles appearing in the dispersions and the T_CP_ values but did not induce particle precipitation. Some amino acids led to a reduction in thermoresponsive polymer aggregate sizes; therefore, they could serve as a replacement for SDS when designing nanocarriers.

The correlation between the transition temperature and the additive used is presented in Figure S10. The acquired data strongly suggest that the influence of additives is not cumulative.

Since the effect of the presence of additives is not straightforwardly synergic, further detailed studies are needed to better understand this phenomenon. Thermoresponsive polymers are frequently proposed as drug carriers; thus, studies of their thermal behavior should be carried out under conditions closely resembling those of physiological and cell culture environments.

## Figures and Tables

**Table 1:** Molar masses and mass dispersity for studied copolymers.

Polymer	M_n_ [g/mol]	M_w_/M_n_
PNIPAM	85,000	1.37
P(HEMA_90_-OEGMA_10_)	66,000	1.45
POEGMA	45,000	1.40

**Table 2:** DMEM components chosen for individual solutions.

DMEM Component	Component Concentration [g/L]	Component Concentration [mol/L]
NaCl	6.4	0.109
NaHCO_3_	3.7	0.044
KCl	0.4	0.005
CaCl_2_	0.2	0.0018
NaH_2_PO_4_	0.109	0.0009
CuSO_4_	0.09767	0.0006
glucose	1	0.0055
L-glutamine	0.584	0.004
L-lysine × HCl	0.146	0.0008
L-izoleucine	0.105	0.0008
L-leucine	0.105	0.0008
L-tyrosine × 2Na	0.10379	0.0004

**Table 3:** Transition temperatures and sizes (highest recorded) of copolymer aggregates formed in water and DMEM.

Polymer	Water		DMEM	
	T_CP_ [°C]	Diameter ^a^ [nm]	T_CP_ [°C]	Diameter ^a^ [nm]
PNIPAM	34.2	215	33.2	770 ^b^
P(HEMA_90_-OEGMA_10_)	22.9	430	20.0	3940 ^b^
POEGMA	70.2	460	64.1	2650 ^b^

^a^ diameter by intensity; ^b^ formation of a precipitate.

## Data Availability

Repository for Open Data: https://doi.org/10.18150/FTLL68.

## Supplementary Materials

The following supporting information can be downloaded at: https://www.mdpi.com/article/10.3390/polym15173587/s1.
