# Supplementary material for: Aggregation of Thermoresponsive Polymethacrylates in a Dulbecco’s Modified Eagle Medium and Its Salts
Source: Polymers (Basel). 2023 Aug 29;15(17):3587. doi: 10.3390/polym15173587 (PMC10489804; doi:10.3390/polym15173587)
Supplement: Supplementary file 1 [file polymers-15-03587-s001.zip › polymers-2576491-supplementary.pdf]

Supplementary information

Aggregation of Thermoresponsive Polymethacrylates in a Dulbecco's Modified Eagle Medium and Its Salts

Łukasz Otulakowski * and Barbara Trzebicka *

Centre of Polymer and Carbon Materials, Polish Academy of Sciences, M. Curie-Skłodowskiej 34, 41-819 Zabrze, Poland

*   Correspondence: lotulakowski@cmpw-pan.pl (Ł.O.); btrzebicka@cmpw-pan.pl (B.T.)

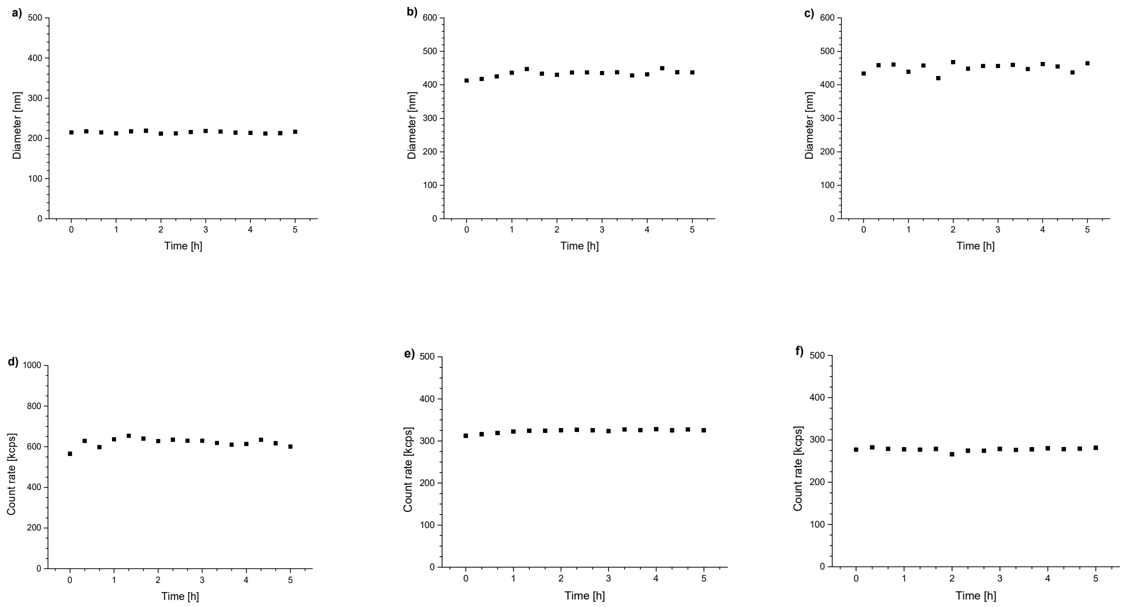

Figure S1. Hydrodynamic diameters of particles vs. time of incubation in water for a) PNIPAM, b) P($HEMA_{90}$-$OEGMA_{10}$), c) POEGMA and scattered light intensity d) PNIPAM, e) P($HEMA_{90}$-$OEGMA_{10}$), f) POEGMA

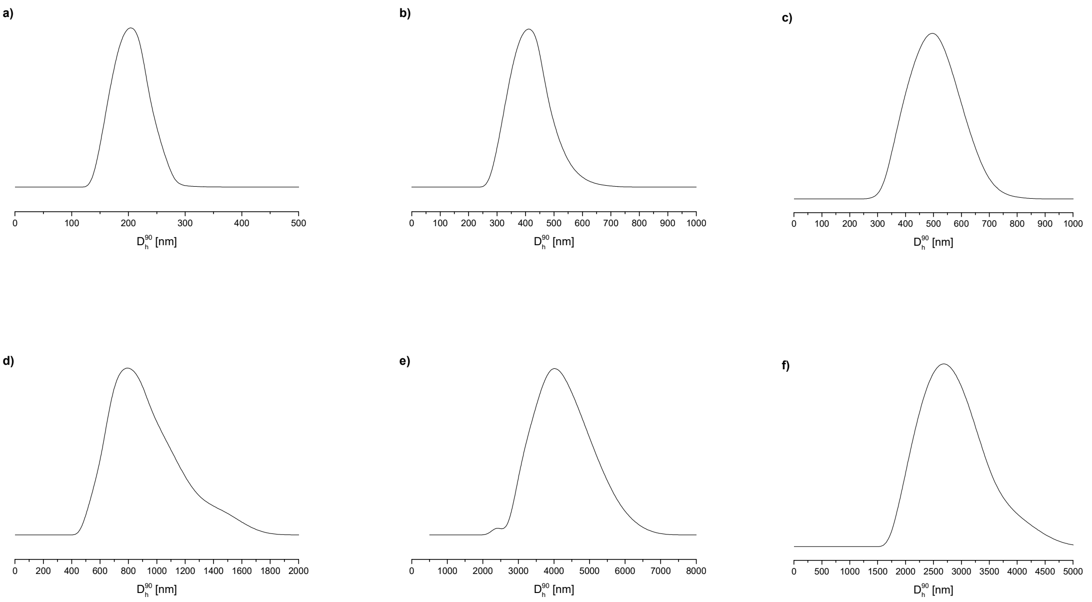

Figure S2. Size distributions of particles formed in water after 5h incubation for a) PNIPAM, b) P(HEMA$_{90}$-OEGMA$_{10}$), c) POEGMA, and size distributions of particles formed in DMEM for d) PNIPAM, e) P(HEMA$_{90}$-OEGMA$_{10}$), f) POEGMA, distributions for highest size detected.

Table S1. DMEM (5030) full composition provided by manufacturer

	**D5030**
**COMPONENT**	g/L
**Inorganic Salts**	
$CaCl_2$	0.2
$Fe(NO_3)_3 \bullet 9H_2O$	0.0001
$MgSO_4$	0.09767
KCl	0.4
$NaHCO_3$	3.7
NaCl	6.4
NaH_2PO_4	0.109
**Amino Acids**	
L-Arginine • HCl	0.084
L-Cystine • 2HCl	0.0626
L-Glutamine	0.584
Glycine	0.03
L-Histidine • HCl • H_2O	0.042
L-Isoleucine	0.105
L-Leucine	0.105
L-Lysine • HCl	0.146
L-Methionine	0.03
L-Phenylalanine	0.066
L-Serine	0.042
L-Threonine	0.095
L-Tryptophan	0.016
L-Tyrosine • 2Na • $2H_2O$	0.10379
L-Valine	0.094
**Vitamins**	
Choline Chloride	0.004

Folic Acid	0.004
myo-Inositol	0.0072
Niacinamide	0.004
D-Pantothenic Acid • ½Ca	0.004
Pyridoxal • HCl	0.004
Riboflavin	0.0004
Thiamine • HCl	0.004
**Other**	
D-Glucose	1.0

Table S2. Results from transmittance and DLS studies for PNIPAM

Component	Component concentration [g/L]	Component concentration [mol/L]	T_{CP} of polymer in solution [°C]	T_{CP} Δ to water	Diameter in 55 °C [by int.]	Diameter Δ to water
Water			34.2		215	
NaCl	6.4	0.109	33.0	-1.2	405*	
NaHCO3	3.7	0.044	33.2	-1	336	121
KCl	0.4	0.005	33.9	-0.3	203	-12
CaCl2	0.2	0.0018	33.9	-0.3	228	13
NaH2PO4	0.109	0.0009	33.3	-0.9	197	-18
CuSO4	0.09767	0.0006	33.8	-0.4	205	-10
Glucose	1	0.0055	34.4	0.2	185	-30
L-Glutamine	0.584	0.004	35.1	0.9	171	-44
L-Lizyne * HCl	0.146	0.0008	34.2	0	184	-31
L-Izoleucine	0.105	0.0008	34.6	0.4	154	-61
L-leucine	0.105	0.0008	34.8	0.6	177	-38
L-Tyrosine *2Na	0.10379	0.0004	34.8	0.6	160	-55
DMEM			33.2	-1	771*	

Table S3. Results from transmittance and DLS studies for $HEMA_{90}$-$OEGMA_{10}$

Component	Component concentration [g/L]	Component concentration [mol/L]	T_{CP} of polymer in solution [°C]	T_{CP} Δ to water	Diameter in 55 °C [by int.]	Diameter Δ to water
Water			22.9		431	
NaCl	6.4	0.109	20.1/31.0	-2.8	1833.6*	
NaHCO3	3.7	0.044	19.9	-3	1881*	
KCl	0.4	0.005	22.3	-0.6	1979*	

CaCl2	0.2	0.0018	22.7	-0.2	1801*	
NaH2PO4	0.109	0.0009	23.0	0.1	416	-15
CuSO4	0.09767	0.0006	22.4	-0.5	597	144
Glucose	1	0.0055	23.1	0.2	432	1
L-Glutamine	0.584	0.004	23.0	0.1	554	123
L-Lizyne * HCl	0.146	0.0008	23.0	0.1	435	4
L-Izoleucine	0.105	0.0008	23.7	0.8	440	9
L-leucine	0.105	0.0008	22.9	0	434	3
L-Tyrosine *2Na	0.10379	0.0004	23.8	0.9	341	-90
DMEM			20.0	-2.9	3942*	

Table S4. Results from transmittance and DLS studies for POEGMA

Component	Component concentration [g/L]	Component concentration [mol/L]	T_{CP} of polymer in solution [°C]	T_{CP} Δ to water	Diameter after 4 h in 55 °C [by int.]	Diameter Δ to water
Water			70.2		462	
NaCl	6.4	0.109	66.6	-3.6	1872*	
NaHCO3	3.7	0.044	68.8	-1.4	2199*	
KCl	0.4	0.005	69.5	-0.7	1126	664
CaCl2	0.2	0.0018	69.6	-0.6	1277	815
NaH2PO4	0.109	0.0009	69.5	-0.7	657	195
CuSO4	0.09767	0.0006	69.4	-0.8	929	467
Glucose	1	0.0055	69.7	-0.5	471	9
L-Glutamine	0.584	0.004	69.5	-0.7	586	124
L-Lizyne * HCl	0.146	0.0008	70.1	-0.1	426	-36
L-Izoleucine	0.105	0.0008	70.0	-0.2	454	-6
L-leucine	0.105	0.0008	70.2	0	420	-42
L-Tyrosine *2Na	0.10379	0.0004	70.7	0.5	543	81
DMEM			64.1	-6.1	2654*	

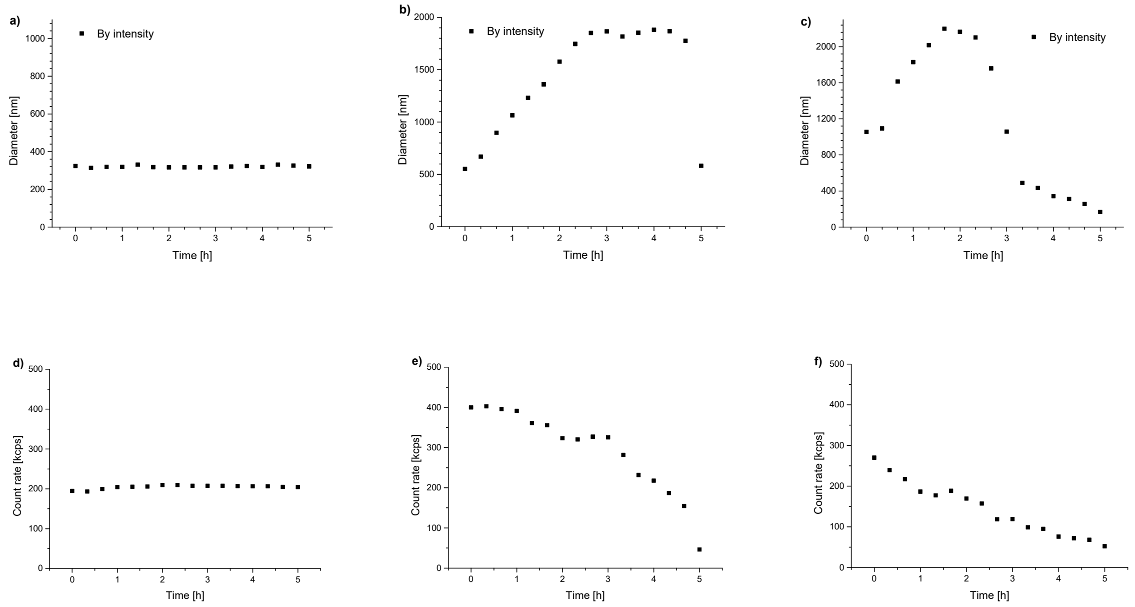

Figure S3. Hydrodynamic diameters of particles vs. time of incubation in solution of $NaHCO_3$ for a)PNIPAM, b) P($HEMA_{90}$-$OEGMA_{10}$), c) POEGMA and scattered light intensity d)PNIPAM, e) P($HEMA_{90}$-$OEGMA_{10}$), f) POEGMA

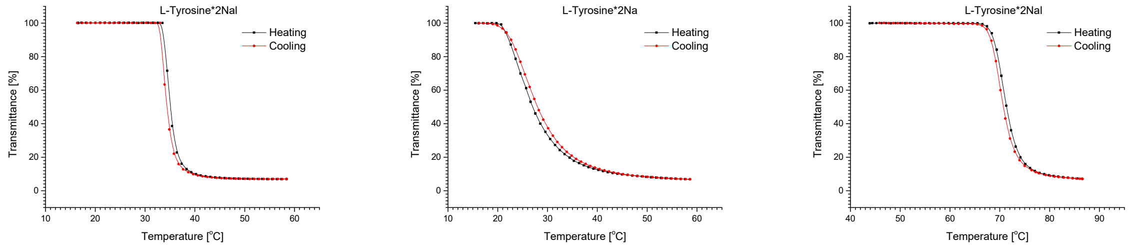

Figure S4. Transmittance vs temperature plots for a) PNIPAM, b) P($HEMA_{90}$-$OEGMA_{10}$), c) POEGMA in solution of L-tyrosine*2Na

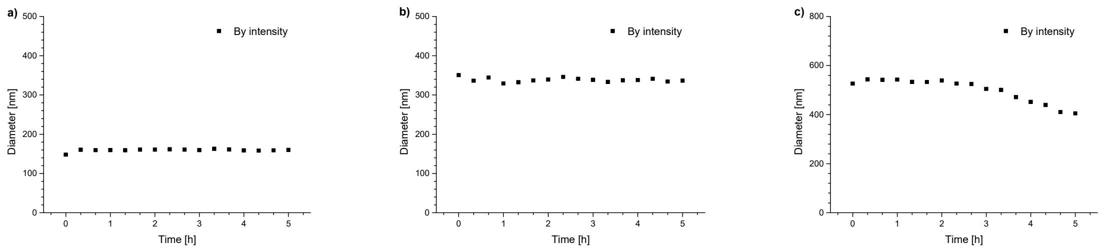

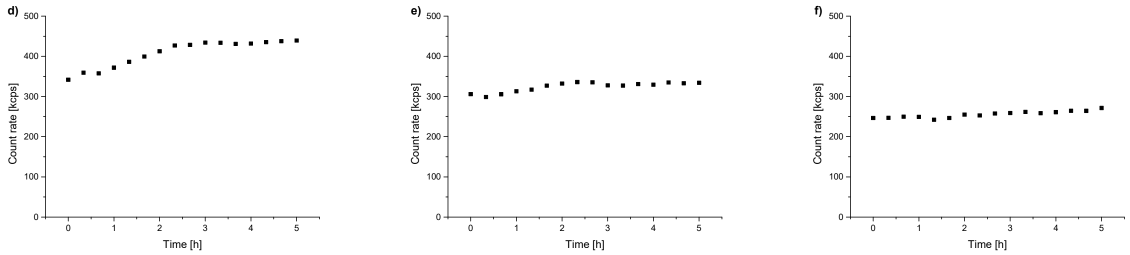

Figure S5. Hydrodynamic diameters of particles vs. time of incubation in solution of L-tyrosine*2Na for a) PNIPAM, b) P($HEMA_{90}$-$OEGMA_{10}$), c) POEGMA and scattered light intensity d) PNIPAM, e) P($HEMA_{90}$-$OEGMA_{10}$), f) POEGMA

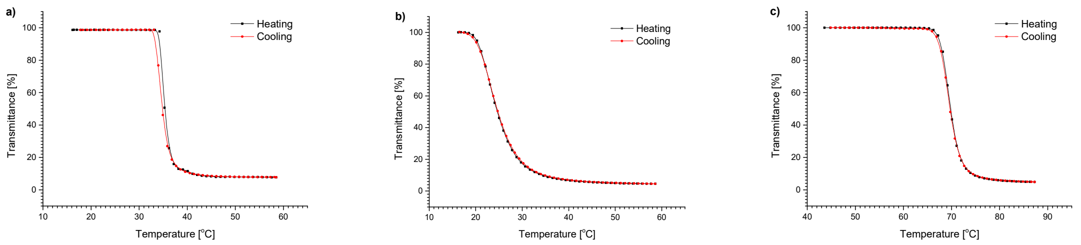

Figure S6. Transmittance vs temperature plots for a) PNIPAM, b) P($HEMA_{90}$-$OEGMA_{10}$), c) POEGMA in solution of L-glutamine.

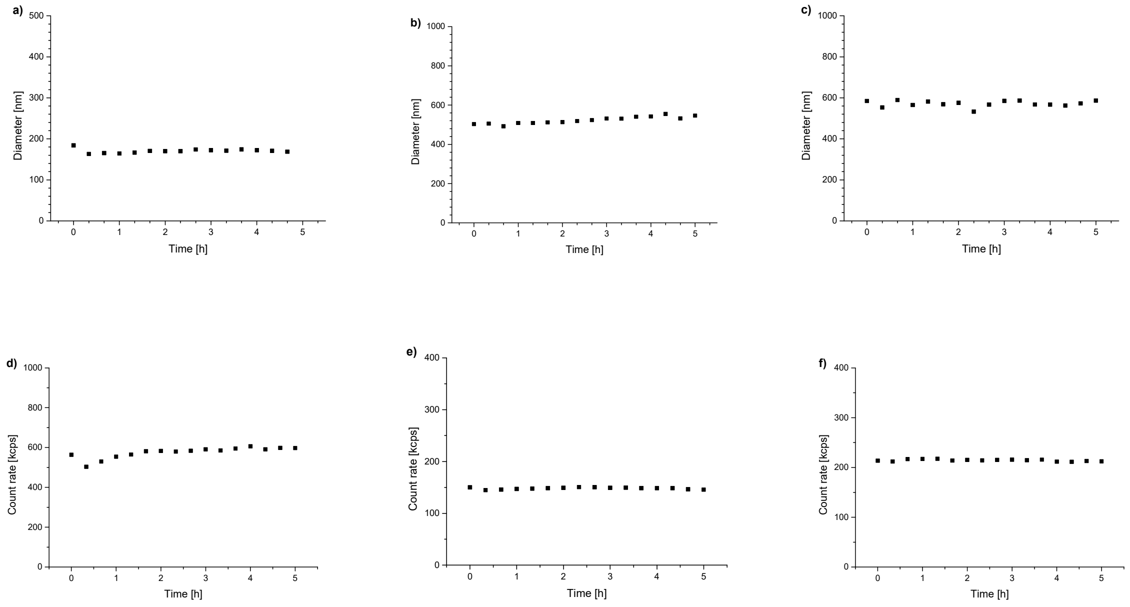

Figure S7. Hydrodynamic diameters of particles vs. time of incubation in solution of L-glutamine for a)PNIPAM, b) P($HEMA_{90}$-$OEGMA_{10}$), c) POEGMA and scattered light intensity d)PNIPAM, e) P($HEMA_{90}$-$OEGMA_{10}$), f) POEGMA

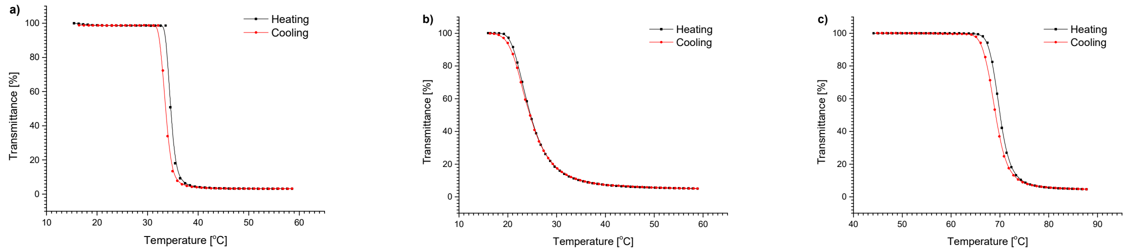

Figure S8. Transmittance vs temperature plots for a) PNIPAM, b) P($HEMA_{90}$-$OEGMA_{10}$), c) POEGMA in solution of glucose

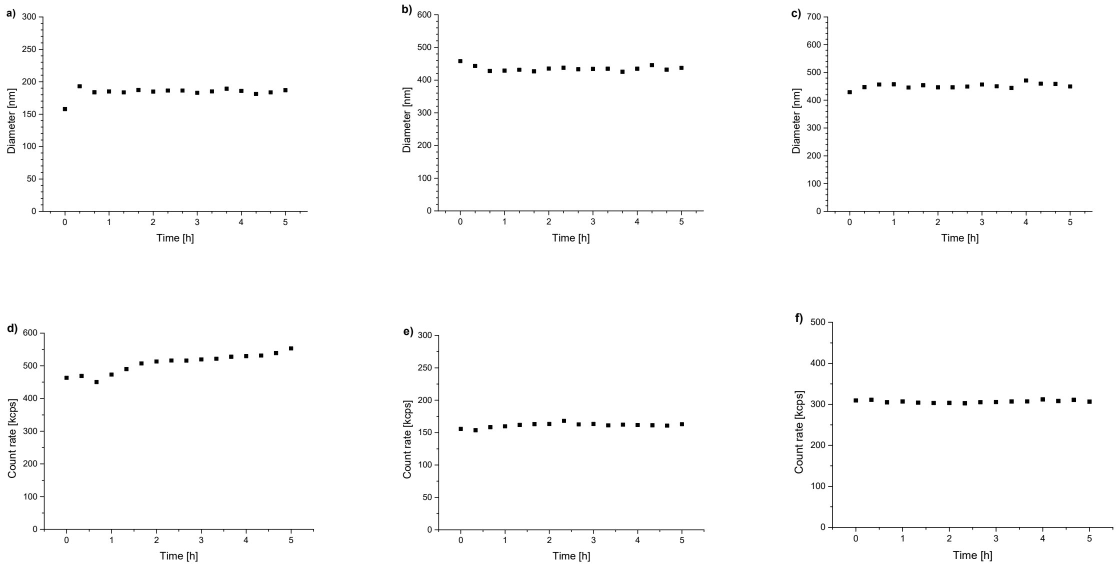

Figure S9. Hydrodynamic diameters of particles vs. time of incubation in solution of glucose for a) PNIPAM, b) P($HEMA_{90}$-$OEGMA_{10}$), c) POEGMA and scattered light intensity d) PNIPAM, e) P($HEMA_{90}$-$OEGMA_{10}$), f) POEGMA

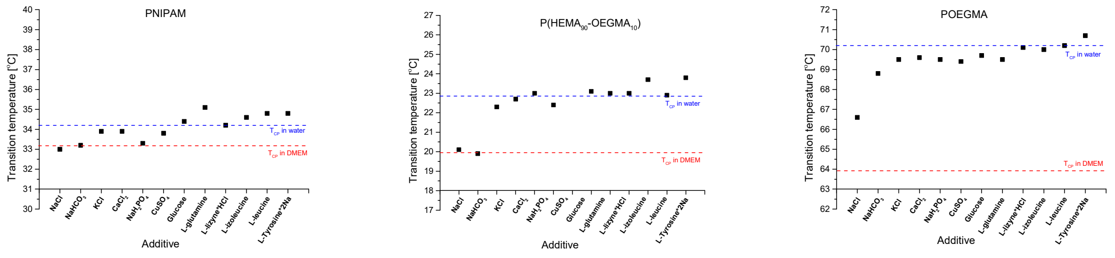

Figure S10. T_{CP} recorded for different additives in comparison with T_{CP} in water and in DMEM for a) PNIPAM, b) P($HEMA_{90}$-$OEGMA_{10}$), c) POEGMA1
